# Golidocitinib was used for the first time to treat refractory NK-Large Granular lymphocytic leukemia with a STAT3 mutation, accompanied by hemolytic anemia: a case report

**DOI:** 10.3389/fimmu.2025.1632803

**Published:** 2025-08-14

**Authors:** Hongyuan Hao, Chenglu Yuan, Chen Zhang, Jimo Jian

**Affiliations:** Department of Hematology, Qilu Hospital of Shandong University, Qingdao, Shandong, China

**Keywords:** golidocitinib, NK-large granular lymphocytic leukemia, refractory granular lymphocytic leukemia, STAT3 mutation, hemolytic anemia

## Abstract

**Background:**

NK-large granular lymphocytic leukemia (NK-LGLL) is a rare clonal lymphoproliferative disease of natural killer (NK) cells. In refractory cases, traditional chemotherapy regimens and immunosuppressive drugs often prove ineffective. Hematopoietic stem cell transplantation (HSCT) offers the opportunity to rebuild the immune system, but carries significant risks, including graft-versus-host disease (GVHD). Consequently, alternative therapeutic strategies need to be investigated. A recent study published in The Lancet reported on the use of golidocitinib in the treatment of relapsed or peripheral T-cell lymphoma. Notably, this represents the first clinical report demonstrating the efficacy of golidocitinib in managing intractable NK-large granular lymphocytic leukemia.

**Case presentation:**

In this study, we present a case of a 53-year-old male with NK-LGLL who achieved successful therapeutic outcomes with golidocitinib administration. The patient had a documented history of chronic gastritis and was admitted with symptoms of fatigue and chest tightness. Despite receiving various treatments such as methotrexate, cyclosporin, cyclophosphamide, and thalidomide, the patient exhibited treatment refractoriness. However, after initiating treatment with golidocitinib, the patient’s blood count improved remarkedly after a single treatment cycle, obviating the need for blood transfusions. The patient maintained golidocitinib treatment without experiencing any serious complications. This is the first reported case demonstrating the efficacy of golidocitinib therapy in treating NK-LGLL.

**Conclusion:**

This case highlights the clinical relevance of golidocitinib in developing novel therapeutic strategies for NK-LGLL. Moreover, this treatment option presents the potential as either a bridging therapy or alternative to allo-HSCT.

## Introduction

Large Granular Lymphoid Leukemia (LGLL) is a rare clonal lymphoproliferative disease of cytotoxic T lymphocytes (CTL) or natural killer (NK) cell. This malignancy constitutes 2-5% of chronic lymphoproliferative diseases (1), and typically exhibits an indolent progression. According to the World Health Organization’s (WHO) 2022 classification, NK-large granular lymphocytic leukemia (NK-LGLL) is classified as a mature NK cell tumor (2). This particular subtype of leukemia demonstrates a more aggressive clinical course, elevated LGL cells in the peripheral blood and hematological abnormalities such as anemia, neutropenia, and thrombocytopenia. Neutropenia, affecting approximately 61% of patients, represents a hallmark feature that significantly increase infection risk, which constitutes the primary cause of disease-related death (3-7%) (3–5). Molecular characterization through identification of mutations in genes including STAT3, STAT5B, TET2, TNFAIP3, and CCL2 provides substantial evidence for supporting the NK cell clonality (6–9). Current first-line immunosuppressive drugs, such as methotrexate, cyclophosphamide, and cyclosporin, demonstrate limited efficacy with complete response rate approximating 16% after 4 months of treatment. Therefore, targeted therapeutic approaches focusing on JAK/STAT pathway and demethylation agent emerge as promising treatment modalities warranting in prospective studies. Golidocitinib is an oral JAK1-selective tyrosine kinase inhibitor. It has demonstrated good anti-tumor activity in clinical trials of relapsed or refractory peripheral T-cell lymphoma. We present a novel case report of a 53-year-old male non-transplant NK-LGLL patient who achieved successful treatment outcomes with golidocitinib.

## Case presentation

**Figure 1:**
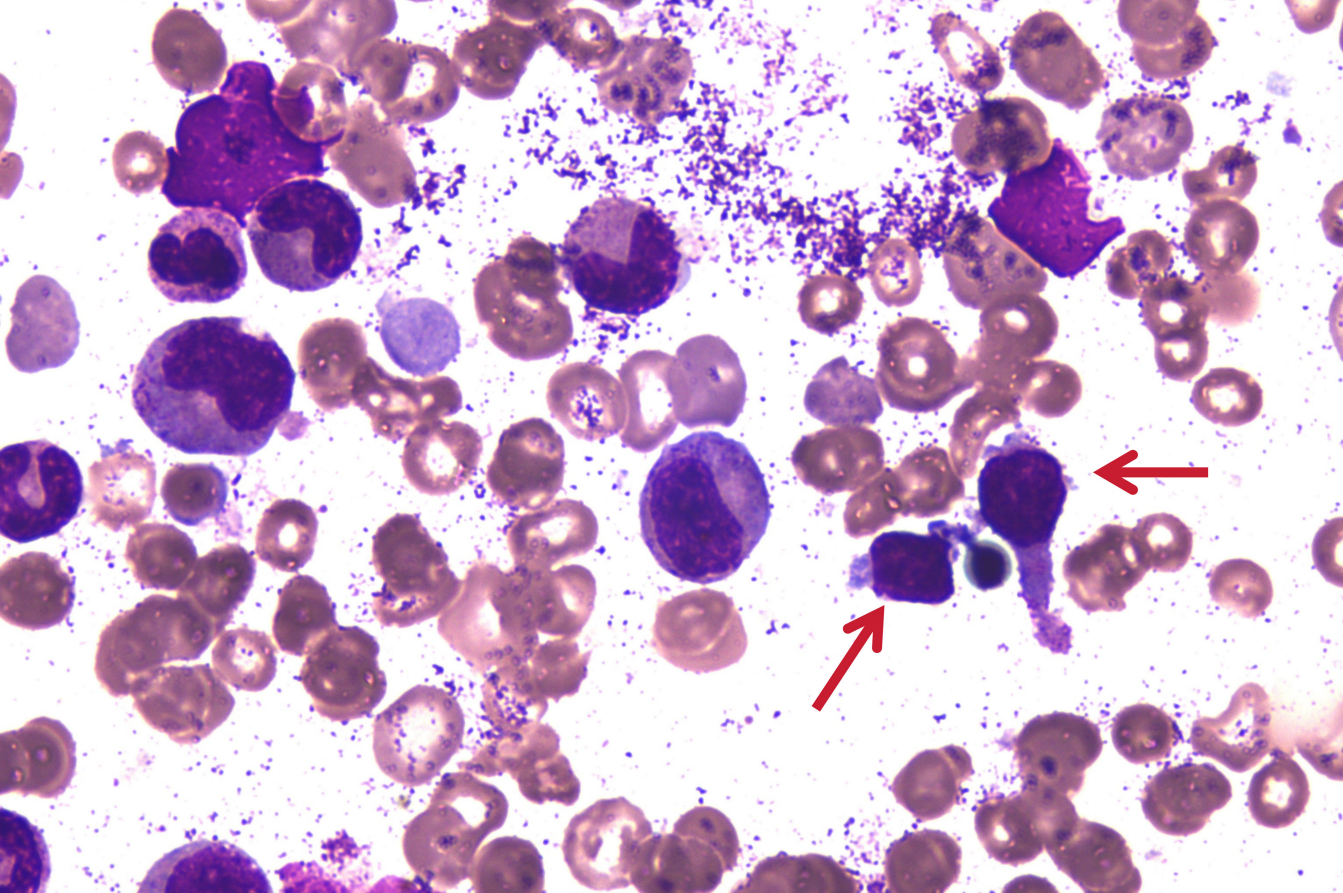
Bone marrow aspiration picture on 24 July 2023. The arrows in the diagram indicate atypical lymphocytes.

In March 2023, a 53-year-old male patient, with no previous history of autoimmune diseases or chronic diseases, initially presented with weakness. His condition progressively deteriorated, culminating in the development of jaundice by July 2023, prompting medical consultation at a hospital. Blood tests revealed a leukopenia (WBC: 3.33×10^9^/L, neutropenia (NEU: 0.55×10^9^/L), anemia (Hb:88 g/L), and thrombocytopenia (PLT: 117×10^9^/L). An ultrasound showed splenomegaly with a thickness of 59 mm and length of 153 mm. The patient was transferred to our hospital, liver function tests indicated abnormal elevations in glutamic oxalacetic transaminase (AST), γ-glutamyltransferase (γ-GT), total bilirubin (TBIL), direct bilirubin (DBIL), and indirect bilirubin (IBIL), accompanied by increased Lactate dehydrogenase (LDH) levels at 755U/L. Bone marrow aspiration revealed an increase in erythroid cells and 7% atypical lymphocytes (**Figure 1**). Bone marrow flow cytometric immunophenotyping identified 22.21% aberrant NK cells, immunophenotype is CD38+, CD16+, CD2+, CD45RA+, CD94+, Perforin, GranzymeB+, mCD3-, TCRg/d-, CD10-, CD57-, CD56-, CD5-, CD4-, CD30-, CD25-, CD26-, CD45RO-, CD161-, cCD3-. KIR series testing revealed diminished CD158 subgroup expression and completed absence of expression of CD159a/CD159c, indicating clonal NK cell proliferation. Next-generation sequencing revealed pathogenic mutations in STAT3 and TET2 genes. In this patient, the clinical meaningful mutation site related to the disease is 2.1% STAT3 p.S614R mutation, which is located in the SH2 domain. The TET2 gene was detected to have the 46.6% TET2 p.E1267Kfs*96 mutation. Histopathological evaluation of bone marrow biopsy confirmed the diagnosis of mature NK cell neoplasm with grade 1 of reticulin fibrosis. Conventional cytogenic analysis showed a normal karyotype [46, XY (20)]. Blood tests revealed elevated indirect bilirubin and 7.53% reticulocytes. It has been further confirmed that the Coombs test was negative. Flow cytometry phenotypic analysis showed that no PNH clones were detected in this patient’s red blood cells, mature granulocytes and mature monocytes. Based on comprehensive diagnostic evaluation, the patient was diagnosed with NK-large granular lymphocytic leukemia complicated by hemolytic anemia.

Patients initiated therapeutic management with a daily dose of 30mg of prednisone and a weekly dose of 17.5mg of methotrexate. Two months later, patient achieved a favorable Hb response of 107g/L. However, subsequent reduction of corticosteroid therapy to 15mg of prednisone daily resulted in recurrent Hb declination of 63g/L. As a result, methotrexate was discontinued and initiation of oral cyclosporine therapy at a dose of 150mg every 12 hours. Additionally, red blood cell transfusions were administered for anemia management. Subsequent monitoring on November 17th, 2023, demonstrated a Hb concentration of 53g/L, accompanied by significant hyperbilirubinemia (TBIL: 74.3μmol/L, IBIL: 42.3μmol/L, DBIL: 32.0μmol/L). Despite these changes, disease progression necessitated further therapeutic escalation, implementing a combination of cyclosporine and COP regimen (cyclophosphamide 1.2g on day 1, vindesine 4mg on day 1, and prednisone 80mg on days 1-5). After the first course of treatment, hematological parameters showed a WBC of 1.49×10^9^/L, a NEU count of 0.48×10^9^/L, a Hb concentration of 81 g/L. The patient subsequently completed two additional COP regimens combined with cyclosporine on December 15, 2023 and January 4, 2024, respectively. After completing three courses of treatment, clinical improvement was evidenced by significant amelioration of fatigue symptoms and normalization of hematological parameters, including Hb concentration (114g/L) and bilirubin levels.

The patient was maintained on a therapeutic regimen of cyclosporine 100mg every 12 hours. However, on April 12, 2024, clinical deterioration was evidenced by the onset of fatigue and laboratory findings demonstrating anemia (Hb: 87g/L) and significant hyperbilirubinemia (TBIL: 81.4μmol/L, IBIL: 34.6μmol/L, DBIL: 46.8μmol/L). This necessitated therapeutic intensification to CHOP regimen (cyclophosphamide 1.2g on day 1, doxorubicin 90mg on day 1, vindesine 4mg on day 1, and prednisone 100mg on days 1-5), administered on April 13, 2024. During the treatment course, the patient developed severe myelosuppression and infection, which were successfully managed with supportive care and antimicrobial therapy. Due to compromised chemotherapy tolerance, the treatment strategy was modified to TPC regimen (thalidomide 50mg qn, methylprednisolone 8mg qd, cyclophosphamide 1.3g qw) on May 20, 2024. However, after treatment, the patient developed choluria and infection, we administrated prednisone, antibiotics, and red blood cell transfusion to manage the hemolytic anemia and infection. While the infection was controlled, hematological recovery remained suboptimal, with a progressively decreasing reticulocyte percentage (0.03%) and persistent anemia (Hb: 51g/L).

On July 11, 2024, the patient underwent comprehensive evaluation due to continued disease progression. Hematological analysis revealed severe cytopenia: leukopenia (WBC: 0.94×10^9^/L), profound neutropenia (NE: 0.13×10^9^/L), anemia (Hb: 30g/L), and thrombocytopenia (PLT: 74×10^9/L). Bone marrow morphology re-examination indicated hypocellularity with a significant reduction in the proportion of granuloid cells and minimal presence of erythroid precursors. Lymphocytic predominance was observed with increased large granular lymphocytes. The immunophenotype of the bone marrow showed 76.3% aberrant NK cells. Histopathological examination of bone marrow biopsy revealed hemorrhagic changes, generally preserved cellularity with erythroid hyperplasia and, along with scattered or clustered lymphocytes, consistent with grade 1 of reticulin fibrosis. Molecular analysis confirmed pathogenic mutations of TET2 and STAT3 genes. These findings collectively indicated progressive clonal NK cell expansion and disease progression. Following thorough discussion of therapeutic options, the patient refused allogeneic hematopoietic stem cell transplantation (allo-HSCT) and instead opted for treatment with golidocitinib, which was approved by the China Food and Drug Administration on June 18, 2024. After providing informed consent, the patient received golidocitinib 150mg once daily orally on July 17, 2024, along with a gradual corticosteroid tapering. After one month of treatment, clinical improvement was evidenced by amelioration of fatigue symptoms and hematological recovery: WBC 2.84×10^9^/L, NEU 2.04×10^9^/L, Hb 65g/L, and PLT 110×10^9^/L. Consequently, the red blood cell infusion was discontinued.

**Figure 2:**
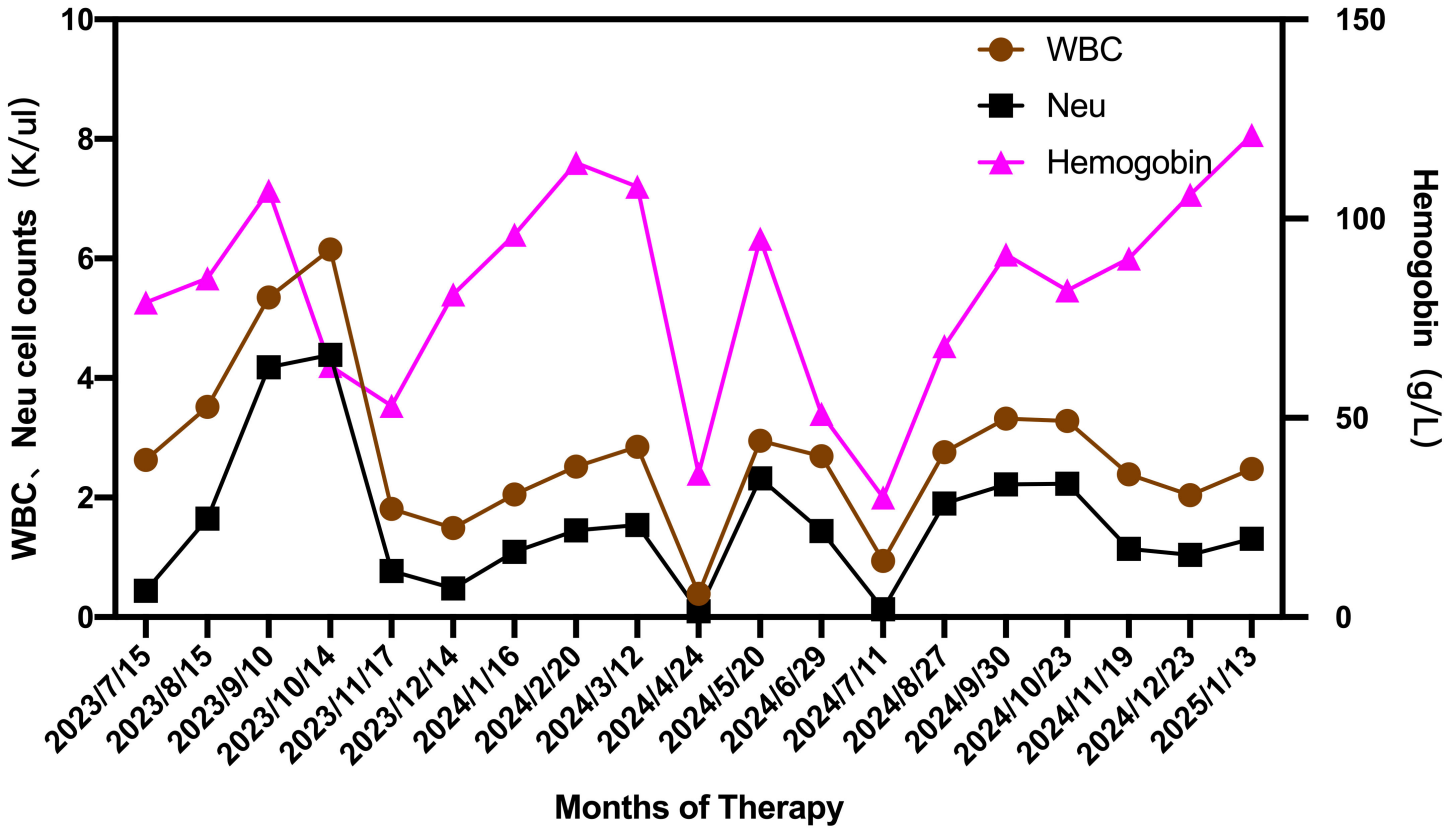
Changes in WBC, NEU and hemoglobin during the therapeutic course from July 2023 to January 2025.

To data, the patient has successfully completed 6-months of golidocitinib treatment. On January 13, 2025, a comprehensive clinical reassessment was conducted. Laboratory findings revealed the following parameters: WBC count was 2.81×10^9^/L, NEU count was 1.41×10^9/L, Hb concentration was 125g/L, and platelet count was 132×10^9^/L (**Figure 2**). Peripheral blood analysis indicated that Large granular lymphocytes accounted for 15% of total lymphocyte population. Hepatic function tests were demonstrated at normal level. Bone marrow morphological examination revealed a 50% increase in erythroid cell proportion. Immunophenotypic analysis of the bone marrow identified 4.19% abnormal NK cells. Genetic testing the conversion of STAT3 to negative status. Pathological examination of bone marrow biopsy indicated generally normal hyperplasia. Abdominal ultrasound showed splenomegaly (thickness: 45mm; Length: 153mm) significant reduction compared to pretreatment measurements. The patient exhibited normalized granulocyte levels, achieved transfusion independence, and demonstrated substantial clinical benefits from the therapeutic.

## Discussion

NK-LGLL is classified as mature NK cell neoplasm (2), characterized by continuous expansion of NK large granular lymphocyte (1, 10). The clinical manifestations of LGLL vary greatly among patients. Neutropenia manifests in 70% to 85% of cases, with 15-39% of patients developing neutropenia-associated infections, which represent the primary cause of mortality in this disease entity. Splenomegaly is observed in 20-50% of cases, while hepatomegaly and lymphadenopathy occur less frequently. Hemolytic anemia and immune thrombocytopenia have been reported in some cases. Anemia is usually moderate, and a small number of patients are dependent on blood transfusion (11). NK-LGLL is pathologically distinguished from reactive or temporary LGL proliferation and the persistence of this clone (4). Current pathological understanding suggests that restricted or complete absent expression of killer cell immunoglobulin-like receptors (KIRs) serves as a reliable indicator of clonal NK cell proliferation (1, 3, 12). The identification of phenotypic restriction patterns in KIRs expression, combined with mutations in STAT3, STAT5B, TET2, TNFAIP3, and CCL2 genes, provides strong molecular evidence supporting NK cell clonality (6). Based on these comprehensive diagnostic criteria, this patient was conclusively diagnosed as NK-LGLL.

Currently, the first-line treatment for NK-LGLL is dominated by cyclophosphamide, methotrexate, and cyclosporin immunosuppressants, which are effective in approximately 50%-60% of patients. While prednisone (1mg/kg once daily) monotherapy can ameliorate cytopenia and alleviate systemic symptoms, it typically fails to induce durable remission (13). In a case report, decitabine, a DNA methyltransferase 1 (DNMT1) epigenetic modulator, demonstrated successful outcomes in a T-LGLL patient refractory to red blood cell transfusion (14). SHP1 is mainly expressed in hematopoietic cells and is associated with DNA methylation. Studies have shown that azacitidine inhibits the activation of STAT3 by restoring SHP1 and down-regulating DNMT1, suggesting that azacitidine as a potential targeted therapy for LGLL (15).

LGLL-related gene mutations exhibit distinct clinical effects. The JAK-STAT signaling pathway is an intracellular signal transduction pathway that is widely expressed in tissues. Specifically, the JAK1-STAT3 pathway has been identified as a critical mediator in T cell lymphomagenesis, promoting tumor cell survival and proliferation (16, 17). Gain-of-function mutations in STAT3 have been found in both T-LGLL and NK-LGLL, providing a unifying pathogenesis for the two subtypes. These mutations are present in up to 60% of T-LGLL cases and approximately 30% of NK-LGLL cases (18). Clinically, STAT3-mutated patients demonstrate stronger cytotoxic characteristics, a higher incidence of neutropenia, autoimmune diseases, a greater treatment requirement, and diminished overall survival (7, 15, 19). TET2 mutations, present in 34% of NK-LGLL cases, correlate with thrombocytopenia and co-occurrence of a other hematologic malignancies (4, 9).

Golidocitinib represents a groundbreaking Class 1 novel drug independently developed in China, marking the first highly selective small molecule inhibitor of JAK1 inhibitor globally approved for relapsed or refractory peripheral T-cell lymphoma. Golidocitinib demonstrates remarkable selectivity, with its affinity for JAK1 exceeding that of JAK2 by more than 214-fold times that of JAK2. This exceptional selectivity enables golidocitinib to precisely target the upstream kinase JAK1 of STAT3, effectively blocking the JAK1-STAT3 pathway while minimizing off-target effects. The clinical efficacy of golidocitinib was evaluated in a Phase II study (JACKPOT8 Part B), which enrolled 104 adult patients with R/R PTCL who had previously received at least one systemic therapy regimen. The study administered a daily dose of 150 mg of golidocitinib over a 21-day cycle. The results demonstrated an ORR of 66.7% in NK/T cell lymphoma with favorable safety profile (20). Following its recognition with “Fast-Track Designation” by the U.S. Food and Drug Administration (FDA) in February 2022. Subsequently, golidocitinib received marketing approval from China’s National Medical Products Administration (NMPA)on June 18, 2024.

In this case study, we present the first reported case of a patient with NK-LGLL who failed to respond to traditional therapeutic agents including methotrexate, cyclosporine, and cyclophosphamide, yet exhibited remarkable clinical improvement with golidocitinib monotherapy. The patient maintained this therapeutic regimen without experiencing any serious adverse events. The encouraging outcomes of this case suggest that golidocitinib warrants further investigation as a potential therapeutic option for LGLL. Additionally, this novel approach may potentially be integrated with allo-HSCT in future treatment strategies. Nevertheless, it is crucial to acknowledge that current follow-up duration remains limited, necessitating extended longitudinal observation to thoroughly evaluate the patient’s long-term prognosis and the duration of response.

## Data Availability

The original contributions presented in the study are included in the article/supplementary material. Further inquiries can be directed to the corresponding authors.
